# Cell membrane sample preparation method of combined AFM and dSTORM analysis

**DOI:** 10.52601/bpr.2022.220004

**Published:** 2022-08-31

**Authors:** Mingjun Cai, Huili Wang, Guanfang Zhao, Hongru Li, Jing Gao, Hongda Wang

**Affiliations:** 1 University of Science and Technology of China, Hefei 230027, China; 2 State Key Laboratory of Electroanalytical Chemistry, Changchun Institute of Applied Chemistry, Chinese Academy of Sciences, Changchun 130022, China; 3 Laboratory for Marine Biology and Biotechnology, Pilot National Laboratory for Marine Science and Technology (Qingdao), Qingdao 266237, Shandong, China

**Keywords:** Atomic force microscopy (AFM), Super-resolution microscopy (SRM), Direct stochastic optical reconstruction microscopy (dSTORM), Cell membrane, Combined technique

## Abstract

A major role of cell membranes is to provide an ideal environment for the constituent proteins to perform their biological functions. A deep understanding of the membrane proteins assembly process under physiological conditions is quite important to elucidate both the structure and the function of the cell membranes. Along these lines, in this work, a complete workflow of the cell membrane sample preparation and the correlated AFM and dSTORM imaging analysis methods are presented. A specially designed, angle-controlled sample preparation device was used to prepare the cell membrane samples. The correlated distributions of the specific membrane proteins with the topography of the cytoplasmic side of the cell membranes can be obtained by performing correlative AFM and dSTORM measurements. These methods are ideal for systematically studying the structure of the cell membranes. The proposed method of the sample characterization was not only limited to the measurement of the cell membrane but also can be applied for both biological tissue section analysis and detection.

## INTRODUCTION

A cell is the smallest unit of life, which is separated from its surroundings by the cell membrane. The cell membrane encloses the entire cell, defines its boundaries and maintains the essential difference between the cytosol and the extracellular environment. The cell membrane is involved in several functions of the cell including cell communication, molecular import and export, cell growth and motility, which are essential for cell survival. The proteins on the cell membrane carry out the majority of the membrane functions and have important physiological roles during the cellular processes. To elucidate the function of the cell membrane, it is quite important to reveal the spatial assembly of the membrane proteins under the implementation of physiological conditions. Therefore, considerable attention has been paid to the high-resolution topographical imaging of both cell membrane proteins and protein complexes.

The atomic force microscopy (AFM), which was invented by Binnig *et al*., is considered to be an important member of the scanning probe microscopy family (Binnig* et al.* 1986). AFM brings numerous advantages to the study of the cell membrane structure, such as easy sample preparation and no need for special treatment like fixation or staining. In addition, the measurements can be carried out without enforcing any imaging conditions like freezing or vacuum. As a result, an accurate and quick image of the cell membrane’s surface and structure of the membrane protein can be obtained with nanometer-based resolution and under most physiological conditions. For that reason, the AFM imaging technology has been utilized for a long in the study of the cell membrane proteins (Jiang* et al.* 2009; Shan* et al.* 2010) and the cell structure (Cai* et al.* 2010; Frederix* et al.* 2009; Goksu* et al.* 2009; Kada* et al.* 2008; Li* et al.* 2017; Wu* et al.* 2009).

Although AFM provides valuable insights from the topography mapping of a sample surface, its imaging capabilities are limited to the surface of the cell membrane. Therefore, a combination of various microscopy approaches has been widely used to expand the application field of AFM (Zhou* et al.* 2017). Fluorescence microscopy is a well-known imaging technique, especially in biological science, which allows the labelling of both intracellular molecules and cellular components with high specificity. The combined imaging analysis approach is mainly focused on the AFM method combined with confocal fluorescence microscopy and total reflection imaging analysis (Frankel* et al.* 2006; Kuyukina* et al.* 2014; Oreopoulos and Yip 2009). However, the resolution of the fluorescence microscopy is two orders of magnitude lower than the AFM, which limits the combination of these two imaging analysis techniques.

The super-resolution microscopy (SRM) belongs to a class of optical imaging technology, which can be divided into two types according to the imaging principle. The first type is based on the illumination field with special intensity distribution, such as the stimulated emission depletion microscopy (STED) and the structure illumination microscopy (SIM). The other is based on single-molecule localization techniques, such as photoactivated localization microscopy (PALM) and direct stochastic optical reconstruction microscopy (dSTORM). The SRM breaks through the optical diffraction limit, providing a favorable analysis pathway for the study of biological samples.

With the development of fluorescence microscopy imaging technology, super-resolution microscopy has increased the resolution of optical microscopy (Huang *et al*. 2009). In addition, super-resolution imaging technology can identify multiple components by employing the multi-color labelling method, which can be used to study and analyze the interaction between different components. In this paper, a sample preparation method of the cell membrane for the simultaneous imaging by both the AFM and dSTORM techniques under near-physiological conditions is introduced. The proposed method can be directly applied to the high-resolution topography imaging of AFM and the molecule localization imaging of dSTORM. Moreover, the proposed method is not limited to the preparation of the cell membrane, but can also be suitable for the study of the biological tissue section.

## EXPERIMENTAL METHODS

### Device for the cell membrane preparation

**Figure 1:**
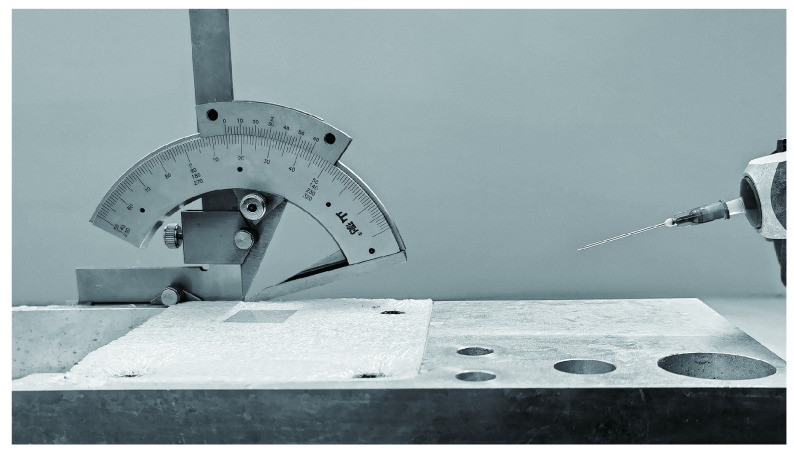
The device for cell membrane sample preparation

The device for the cell membrane preparation was custom-built for erythrocytes (red blood cells) and cultured cells (Fig. 1). A 10-mL syringe was used as a liquid injection device. The injection angle was adjusted by an angle meter. The cells were adsorbed on the glass coverslip (22 mm × 22 mm, Thermo Scientific) and the glass coverslip was put on the pedestal of the device. The water flow was ejected from the syringe and impinged on the surface of the coverslip. Moreover, the proposed device configuration can be fixed on the laboratory bench.

### Preparation of the APTES-coverslips

**Figure 2:**
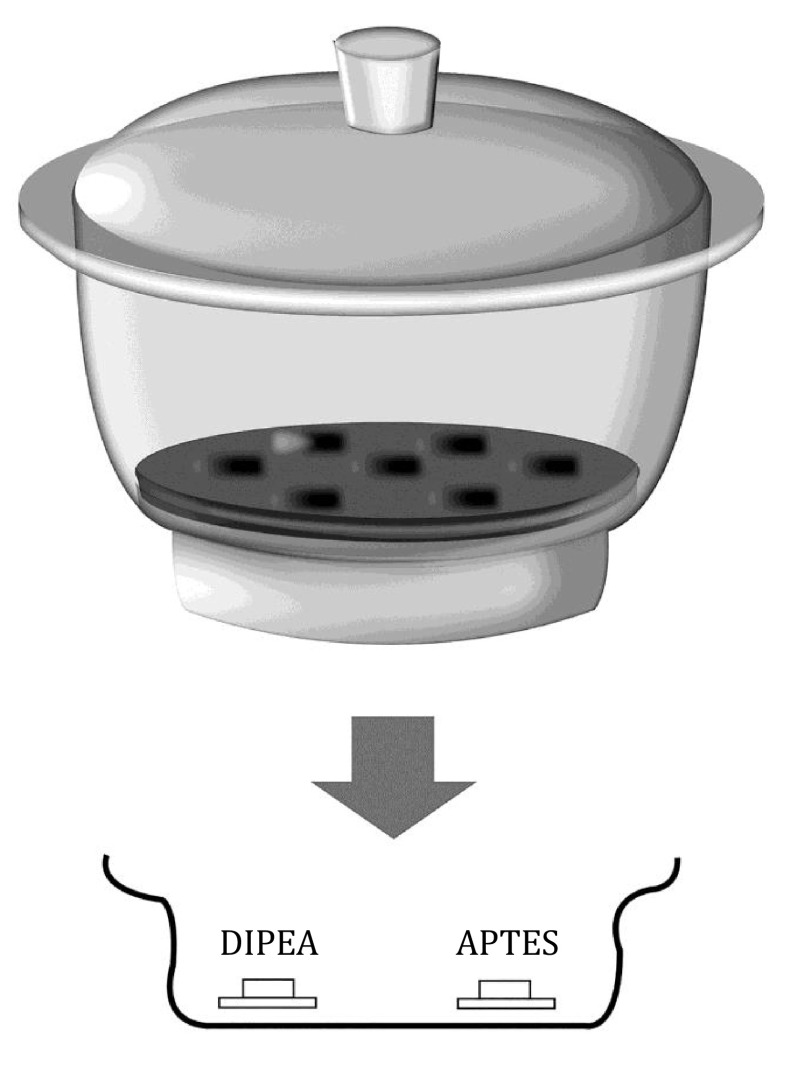
Schematic diagram of the preparation process of the APTES-coverslip. The reagents DIPEA and APTES were placed in small containers on the bottom of the desiccator. Silylation solution: 3-aminopropyltriethoxysilane (APTES); N,N-diisopropylethylamine (DIPEA)

The device for producing the APTES-coverslip is shown in Fig. 2. Firstly, two small dishes were placed in a clean desiccator, while then several clean and dry coverslips were evenly placed on the small dishes. Afterward, argon gas was introduced and kept for 5 min to empty the air and completely the desiccator with argon gas. 50 μL of APTES (3-Aminopropyltriethoxysilane, Sigma) and 15 μL of DIPEA (N,N-diisopropylethylamine, Sigma) were added to the two small dishes, and the desiccator was again filled with argon gas for 5 min. Subsequently, the desiccator was sealed and kept at room temperature for 4 h. After that, the silylation reaction was finished and the APTES-modified coverslips can be used. The silanized coverslips can be stored in the sealed desiccator for one week, while the argon gas needs to be reintroduced every time the coverslips are removed.

### Preparation of the cytoplasmic side of cell membranes by sheared-open methods

**Figure 3:**
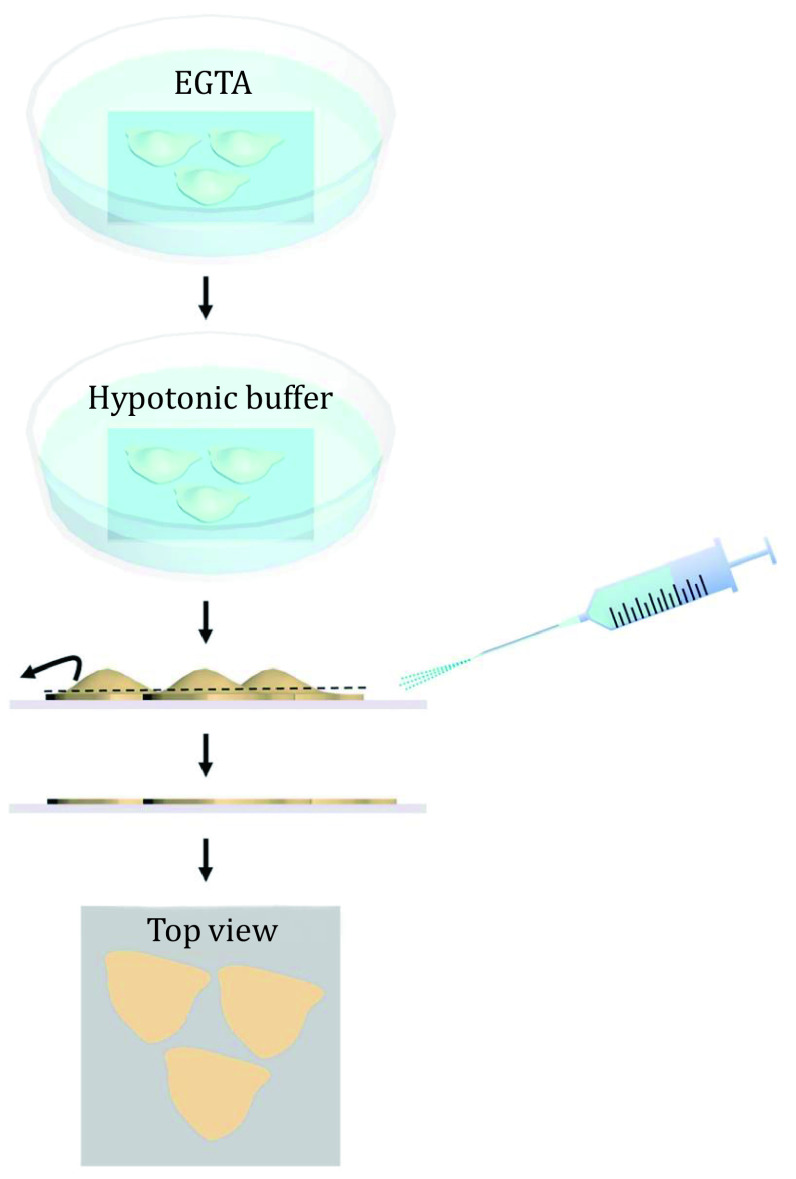
Schematic illustration of the workflow for preparing the cytoplasmic side of cell membranes by using the sheared-open method

The 16HBE cell line (human bronchial epithelium) was used in this protocol. To obtain the cytoplasmic side of the cell membrane, the detached cells were incubated for 15 min on APTES-coverslips and then were cultured in the medium for two days. As shown in Fig. 3, the cells were firstly incubated in ice-cold PBS buffer (150 mmol/L NaCl, pH 7.5) containing 2 mmol/L EGTA for 2 min to separate the tight junctions, followed by washing the cells with PBS buffer. Subsequently, they were incubated in hypotonic PBS buffer (7.5 mmol/L NaCl, pH 7.5) for 3 min on ice and the cells were sheared open by a rapid stream of 8 mL PBS hypotonic buffer through a needle at an angle of 20°. To remove the membrane skeletons, the sheared open membranes were incubated in low-salt buffer (6.85 mmol/L NaCl, 0.135 mmol/L KCl, 0.075 mmol/L KH_2_PO_4_, and 0.405 mmol/L Na_2_HPO_4_·12H_2_O, pH 7.2) for 10 min on ice. Finally, the cytoplasmic side of the membranes was washed with PBS buffer and stored in PBS buffer at 4 °C.

For only the AFM imaging, the prepared membranes could be directly imaged. To combine the AFM imaging technique with the dSTORM, the prepared membranes were initially immersed in a 1% (*w*/*v*) paraformaldehyde (PFA) solution for 15 min and washed with PBS buffer three times. Then, the membranes were incubated in 4% (*w*/*v*) bull serum albumin (BSA) for 30 min. Subsequently, the cell membranes were stained with the fluorescence dyes in dark for 1 h, and washed three times with PBS buffer to remove excess dyes. At this time, AFM imaging could be performed. After the image acquisition, a PBS buffer solution containing 100 nm TetraSpeck microspheres (Invitrogen) was added into the sample for correction of the *x*-*y* drift. Then the sample was washed with PBS buffer and dSTORM imaging buffer was added. It should be noted that the AFM imaging is usually performed before adding the dSTORM imaging buffer since the latter contains enzyme oxygen scavenger, which is not compatible with the AFM imaging.

### Image acquisition on the correlative dSTORM/AFM microscopy

**Figure 4:**
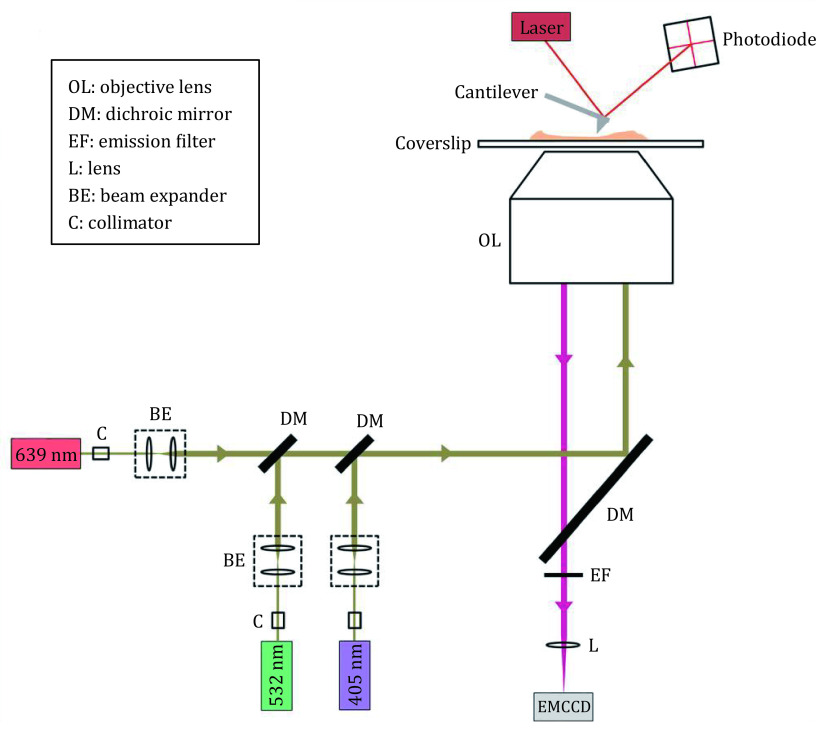
Schematic diagram of the combined AFM and dSTORM technique

The correlative SRM and three-dimensional (3D) topography imaging microscopy methods were carried out by combining an AFM and an inverted fluorescence microscopy (Fig. 4). For the AFM imaging, the respective topographic images were recorded in an acoustic AC mode by using oxide-sharpened Si_3_N_4_ probes (DNP-S, Veeco, USA) with a nominal spring constant of 0.06 N/m at a scanning speed of 1.0 Hz. All the acquired images were recorded at 512 × 512 pixels. The dSTORM imaging was conducted under total internal reflection fluorescence (TIRF) illumination with an oil-immersion objective (100× 1.49 NA, Nikon, Japan), appropriate optical filters (dichroic: ZT405/488/532/640rpc-XT; emission filter: ZET532/640m, Chroma) and EMCCD camera (iXon Ultra 888, Andor). For the single-color imaging, the samples were excited with a 639 or 532-nm laser (intensity of ~2 W/cm^2^), while being concurrently activated with a 405-nm laser (0–1 W/cm^2^). The dual-color imaging process was conducted in a similar way as the single-color imaging except for excitation, which was sequentially performed by a 639-nm (Alexa647) and a 532-nm laser (Alexa532). For the dual-color imaging, the two-wavelength channels were further filtered by FF01-559/34 (Semrock) bandpass filters in the short wavelength and ET700/75m (Chroma) in the long-wavelength channel. In this work, 10,000 frames with a 20-ms exposure time were recorded and the electron gain of 300 was attained to construct the super-resolution images. During the acquisition time, the *z*-drift was eliminated by employing a focus lock. As far as cell membrane preparation is concerned, multi-color microspheres were embedded as fiducial markers to correct the lateral drift and to calibrate chromatic aberration for the dual-color imaging.

The raw image sequences were processed with the ThunderSTORM software, which determined the subdiffraction localizations by fitting a suitable point spread function (PSF) model and using the maximum-likelihood estimation approach. To eliminate localizations with a too dim or wide value of PSF, localizations with a precision higher than 30 nm and a photon count of more than 100 were considered. Besides, the appearance of localizations in consecutive frames within 20 nm was merged into a single localization. The reconstructed dSTORM images were viewed based on an average-shifted histogram approach.

### Registration between AFM and dSTORM

#### Registration between low-resolution AFM topography and dSTORM images

**Figure 5:**
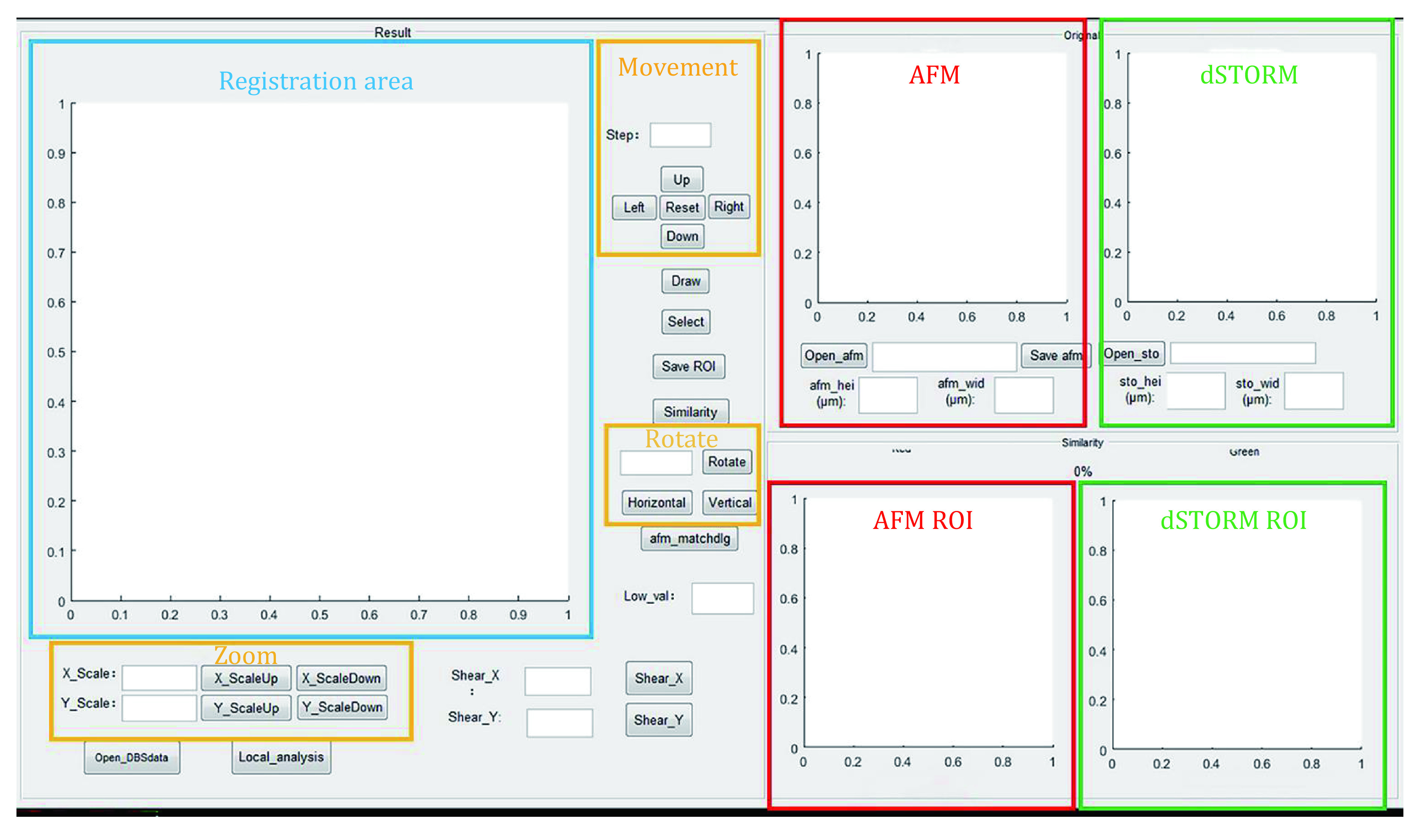
Depiction of the custom MATLAB program with a GUI to achieve registration between the AFM and the dSTORM images

According to the described matching algorithm between AFM and dSTORM images, a custom-written MATLAB program (CorrImgReg.m) with a graphical user interface (GUI) was utilized to achieve registration between AFM and dSTORM images (Fig. 5). Firstly, the acquired AFM topography and the corresponding dSTORM image were imported. Since various aberrations are involved in both AFM and dSTORM imaging and a larger field of view than AFM images is usually contained in the dSTORM images, registration for the low-resolution AFM topography and dSTORM image was performed by employing affine transformations, on top of that, the transform parameters were adjusted by optimizing the overlap between the structures in the AFM and dSTORM images. The used two-dimensional (2D) affine transformation is defined based on Eq. 1 and Eq. 2.

1 $ x' = Ax + By + Tx ,    $

2 $ y' = Cx + Dy + Ty ,   $

where *x* and *y* are the original coordinates (dSTORM localizations), *x*' and *y*' stand for post-transformed coordinates, *A*, *B*, *C* and *D* represent the lumped parameters that can cause rotation, scale and shear operations, respectively, whereas *Tx* and *Ty* denote the translation parameters. According to the sum of the post-transformed dSTORM localizations with a signal in the corresponding area of the AFM image, accurate registration of low-resolution AFM topography and dSTORM images is achieved.

#### Registration between low and high-resolution AFM topography

**Figure 6:**
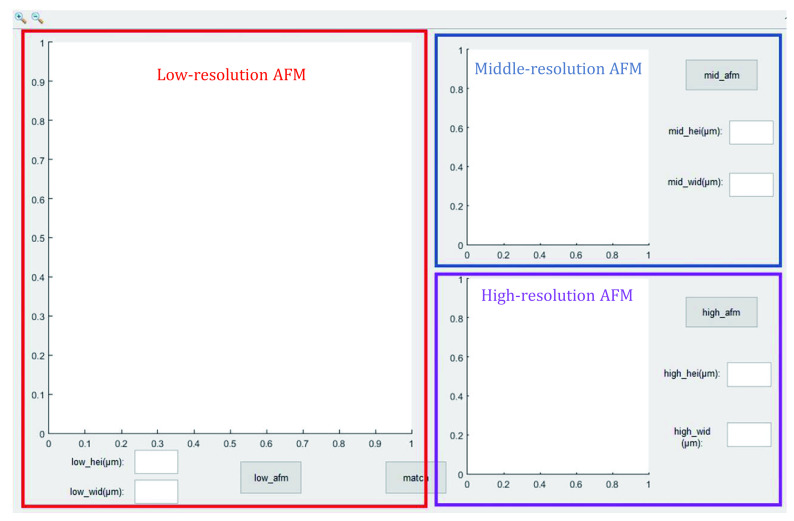
Depiction of the custom MATLAB program with a GUI to achieve registration between the low- and high-resolution AFM topography

Accurate registration of the high-resolution AFM topography in the corresponding low-resolution AFM topography is achieved by employing another custom-written MATLAB program (afm_match.m) with a GUI (Fig. 6). Firstly, low-, medium- and high-resolution AFM topography of the same cell membrane were imported separately. Then, the position of the high-resolution AFM topography can be detected in the corresponding low-resolution AFM topography by the cross-correlation method in the program. Consequently, the exact registration between the low- and high-resolution AFM topography can be achieved.

#### Registration between the high-resolution AFM topography and the corresponding area in the dSTORM image

The precise position of the high-resolution AFM topography in the low-resolution AFM topography can be read by the previous MATLAB program (CorrImgReg.m) and the same region in the corresponding dSTORM image can be extracted. Finally, precise registration between the high-resolution AFM topography and the target region in the dSTORM image can be achieved.

## RESULTS

**Figure 7:**
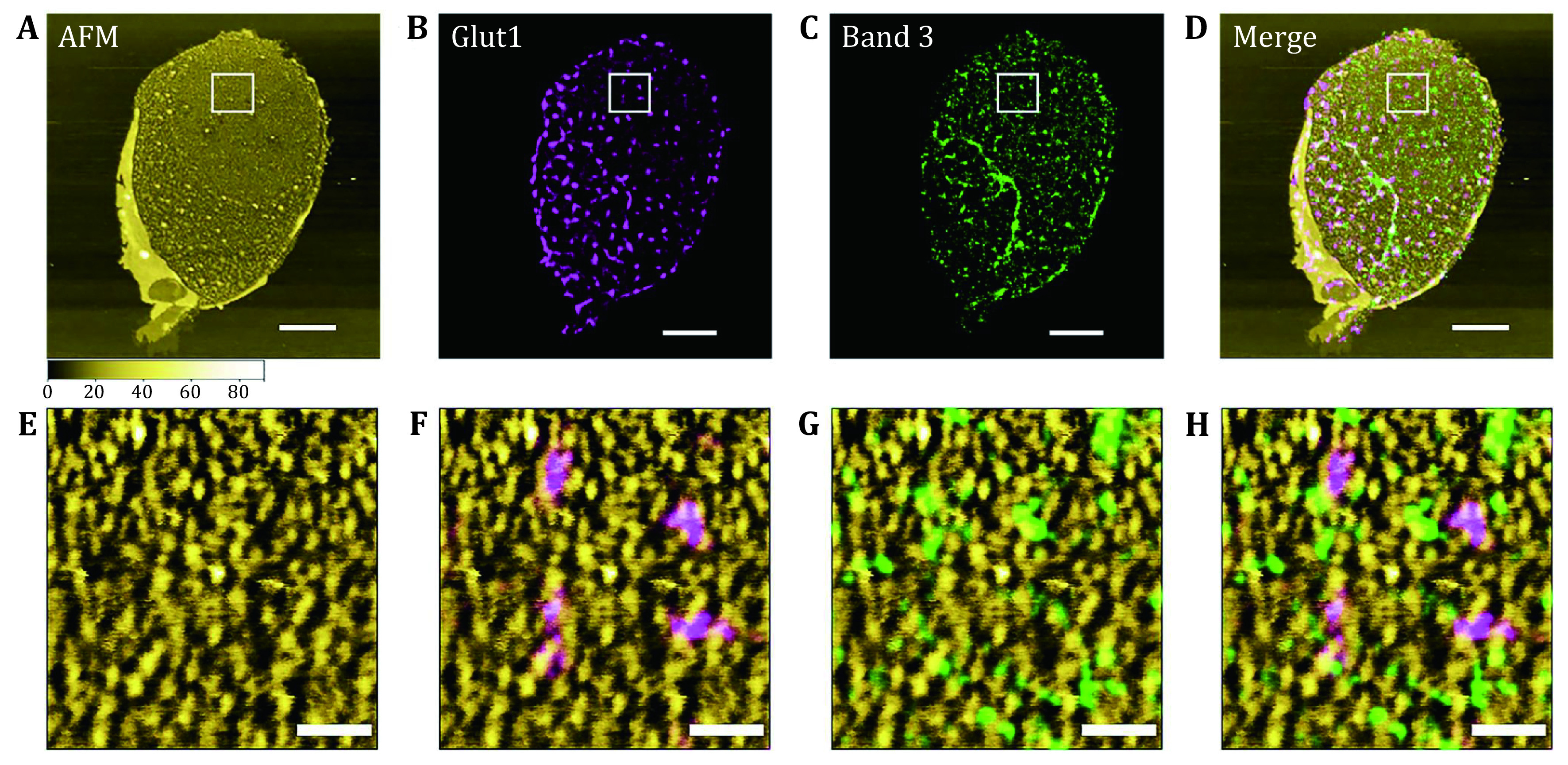
Identification of Glut1 and Band 3 in the topography of the cytoplasmic side of the erythrocyte membrane. **A** Topography of the cytoplasmic side of the erythrocytes membrane. **B**, **C** Distribution of Glut1 and Band 3 in the same erythrocyte membrane. **D** Merged image of Panels A−C where the locations of Glut1 and Band 3 are identified in the topography of the cytoplasmic side of the erythrocyte membrane. **E** High-resolution topography of the corresponding area (white box in Panel A). **F**–**H** Localization of Glut1 and Band 3 in the high-resolution topography, respectively and the merged image. Scale bars: 3 μm (Panels A–D) , 300 nm (Panels E–H)

A typical experiment combines the related dSTORM/AFM analysis techniques, which can be used to detect the location of specific proteins on the cell membrane and reveal the nanostructure of the protein complexes. Mature mammalian erythrocytes do not have a nucleus, so the plasma membrane of the erythrocytes is the simplest and easiest biological membrane to study. Once the plasma membrane of an erythrocyte is ruptured by hypoosmotic treatment, the hemoglobin and other intracellular soluble proteins are released and the erythrocyte membrane retains its original shape and size. Therefore, erythrocytes are ideal to investigate the plasma membrane structure. By combining the AFM and the dual-color dSTORM methods we can draw the conclusion that the protein clusters of glucose transporter 1 (Glut1) and band 3 protein (Band 3) were mainly located in the protein islands of the topography. In addition, the protein islands in topography also interacted with each other to assemble into larger protein clusters or functional microdomains (Fig. 7) (Zhao* et al.* 2021).

**Figure 8:**
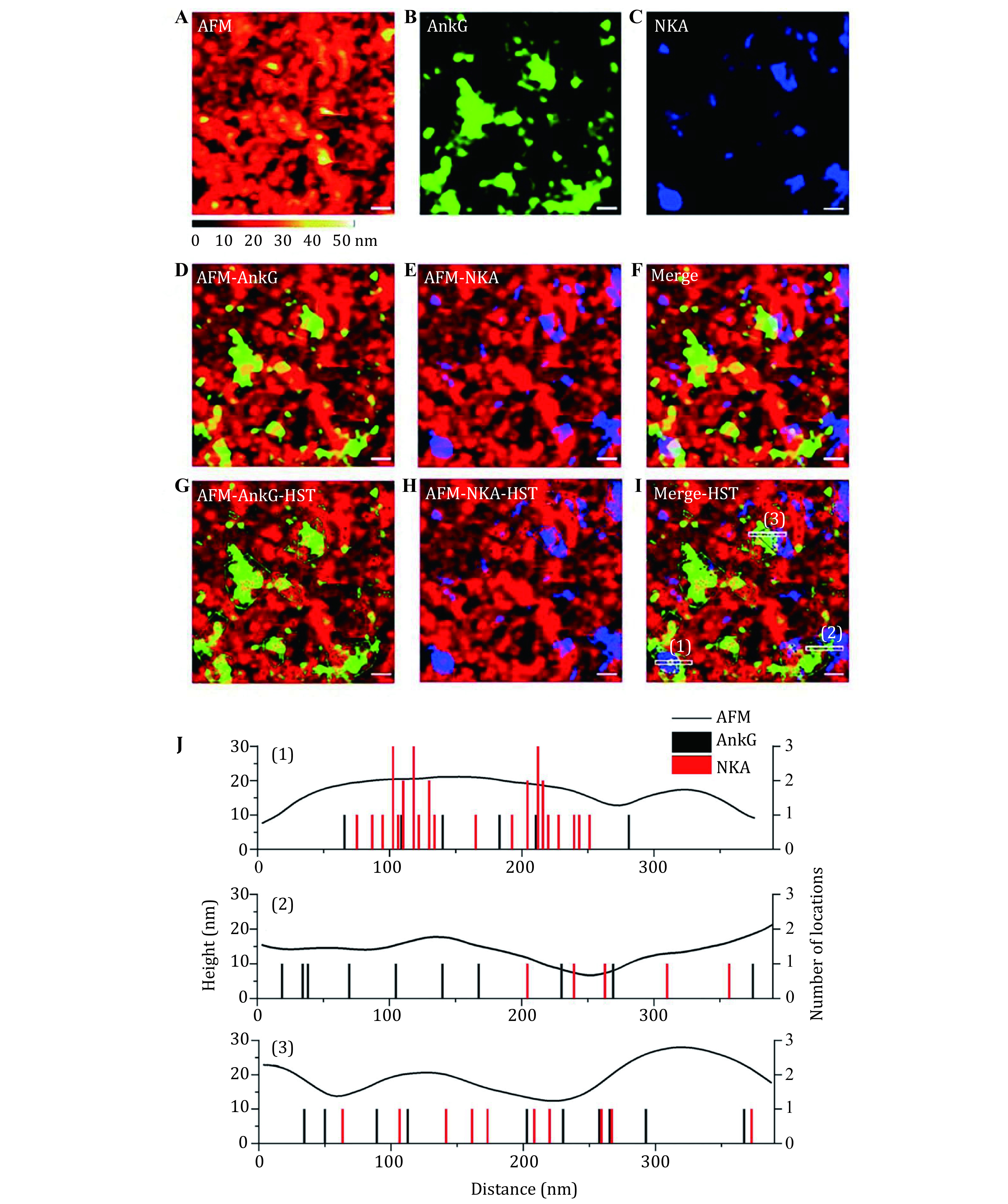
Localization of AnkG and NKA in the high-resolution cell membrane morphology images. **A**–**C** The correlative high-resolution AFM image and dual-color dSTORM images of AnkG and NKA. **D**–**E** The merged image of AnkG and NKA within the membrane topography. **F** The merged image of Panels A–C. **G**–**I** Depiction of clusters of the specific proteins on the correlative maps. The green (blue) dots represent the true locations of each AnkG (NKA) molecule and green (blue) polygon boxes circle the clusters of AnkG (NKA) molecules. **J** The correlation analysis among AFM height, AnkG and NKA positions in the white barred region of Panel I

In addition, important membrane proteins such as Na^+^–K^+^ ATPase (NKA) and ankyrin-G (AnkG) of the mammalian cells were explored by this method. More specifically, the observation on the cytoplasmic side of the 16HBE cell membrane revealed that these two kinds of membrane proteins might exist as clusters to perform their physiological functions. To further observe the distribution of these clusters in the membrane topography, correlative high-resolution AFM and dual-color dSTORM images were performed. The extracted results confirmed that the clusters were protein islands composed of identical proteins (Fig. 8). Overall, the nano-organization and localization of NKA and AnkG, as well as the interaction between NKA and AnkG in the membrane topography provide useful insights into elucidating the transmembrane linkage existing between the cytoskeleton and the cell membrane (Zhou* et al.* 2020b).

## DISCUSSION

APTES can modify a layer of amino groups on the coverslip surface, unlike the conventional cell apposition, where active ammonia can couple to biomolecules such as proteins and DNA, thus allowing the cell membrane to be tightly adsorbed to the modified coverslip substrate. We have to underline that the surface of coverslips without APTES modification was also examined. The acquired results showed that the cell membranes were not tightly attached and as a result, they can be easily detached. Furthermore, this approach also carefully preserves the native ultra-structure upon which such hypotheses are based and enables cell-wide/cell-to-cell investigation of the natural variability in protein-ultrastructure relationships. With the advent of combined imaging by super-resolution microscopy and AFM, a closer resolution between the two models can be obtained. In addition, the information on the morphology and the spatial location of the specific membrane protein molecules can be displayed with nanoscale resolution in a crowded environment of the cell membrane.

The combination of the two techniques can also complement each other in the study of cell membrane structure, protein distribution and localization, *etc*. For biological ultrastructure, the structural information of the single-molecule localization and 3D morphology of biomolecules can be explored simultaneously. Moreover, the structural and kinetic information of biomolecules can be further investigated based on single molecule force spectroscopy (Zhang* et al.* 2021; Shan and Wang 2015). The combined technology can also promote the development of nano-detection in the field of biomedicine (Chacko* et al.* 2014; Pi* et al.* 2014). A combination of techniques will significantly promote the application of single molecule technologies in the biological field (Muller* et al.* 2021; Nandi and Ainavarapu 2021; Odermatt* et al.* 2015). Interestingly, the application of super-resolution microscopy combined with atomic force microscopy apparatus and method will have wide application prospects, not only in the field of biological imaging, but also in materials science, biosensor and other fields (Hauser* et al.* 2017).

At present, AFM and fluorescence microscopy are combined for biological studies, while the resolution of conventional optical imaging is two orders of magnitude lower than that of AFM imaging due to the existence of the optical diffraction limit. In addition, due to the limitation of both the equipment and the resolution, these two imaging techniques are mostly purely superimposed and lack data integration. Therefore, the joint data of the related imaging devices cannot be obtained based on a single imaging modality. Thus, the co-localization resolution of the AFM morphology information and super-resolution imaging information was calibrated through data conversion and coordinate conversion to achieve the true superposition of the two data. The platform can analyze the overall 3D morphology and the specific components of the biological samples with nanometer resolution, and correlate the two sets of data at the same time (Zhou* et al.* 2020a).

The platform is based on the application of atomic force microscopy and super-resolution microscopy, which allows for the implementation of both *in situ* imaging and localization analysis of proteins on the cell membranes. The two imaging techniques are combined to capture structural information of cell membrane proteins under near physiological conditions. At the same time, due to the fast and reliable sample preparation method, the physiological structure of the sample can be preserved to a large extent, rendering thus our approach suitable for studying both the cell membrane structure and the underlying function. Furthermore, it can be used for cell and extracellular matrix imaging analysis. The combination of these two imaging techniques in live cell analysis has also yielded encouraging results, in terms of interactions between virus and live host cell. The collection of these multimodal cross-scale experimental data is of great importance for the execution of deep learning based artificial intelligence computations to explore the biomembrane functional networks.

## CONCLUSIONS

The AFM imaging and mechanical analysis in conjunction with the single-molecule recognition imaging based on AFM were carried out on erythrocyte membrane, nucleated cell membrane and organelle by the sheared open method. With the development of super-resolution and atomic force microscopy techniques, a closer spatial resolution between the two modes can now be obtained. Therefore, specific membrane protein molecules with nanoscale resolution can be visualized in a crowded environment of the cell membrane. The correlative SRM and AFM analysis can elucidate the details of the cell membrane ultrastructure by directly visualizing the nanoscale relationship of the specific proteins. In short, the proposed hyphenated technique could help in understanding the nanoscale organization of the cell membrane and the relationship between the membrane and thousands of proteins.

## Conflict of interest

Mingjun Cai, Huili Wang, Guanfang Zhao, Hongru Li, Jing Gao and Hongda Wang declare that they have no conflict of interest.
